# An Efficient Partition-Based Approach to Identify and Scatter Multiple Relevant Spreaders in Complex Networks

**DOI:** 10.3390/e23091216

**Published:** 2021-09-15

**Authors:** Jedidiah Yanez-Sierra, Arturo Diaz-Perez, Victor Sosa-Sosa

**Affiliations:** 1 CINVESTAV-Guadalajara, Guadalajara 45019, Mexico; adiaz@cinvestav.mx; 2 CINVESTAV-Tamaulipas, Ciudad Victoria 87138, Mexico; vjsosa@cinvestav.mx

**Keywords:** complex networks, spreaders selection, communities

## Abstract

One of the main problems in graph analysis is the correct identification of relevant nodes for spreading processes. Spreaders are crucial for accelerating/hindering information diffusion, increasing product exposure, controlling diseases, rumors, and more. Correct identification of spreaders in graph analysis is a relevant task to optimally use the network structure and ensure a more efficient flow of information. Additionally, network topology has proven to play a relevant role in the spreading processes. In this sense, more of the existing methods based on local, global, or hybrid centrality measures only select relevant nodes based on their ranking values, but they do not intentionally focus on their distribution on the graph. In this paper, we propose a simple yet effective method that takes advantage of the underlying graph topology to guarantee that the selected nodes are not only relevant but also well-scattered. Our proposal also suggests how to define the number of spreaders to select. The approach is composed of two phases: first, graph partitioning; and second, identification and distribution of relevant nodes. We have tested our approach by applying the SIR spreading model over nine real complex networks. The experimental results showed more influential and scattered values for the set of relevant nodes identified by our approach than several reference algorithms, including degree, closeness, Betweenness, VoteRank, HybridRank, and IKS. The results further showed an improvement in the propagation influence value when combining our distribution strategy with classical metrics, such as degree, outperforming computationally more complex strategies. Moreover, our proposal shows a good computational complexity and can be applied to large-scale networks.

## 1. Introduction

Networks are being increasingly used for representing, analyzing, and explaining complex systems. According to Gartner, graph analysis can efficiently model, explore, and query data with complex interrelationships across data silos, and it will grow at 100 percent annually through 2022 to continuously accelerate data preparation and enable more complex and adaptive data science [1]. A key area in the network science field is related to the study of information spreading in a network [2].

Identification of influential nodes (also called propagators, relevant nodes, or vital nodes) that can help accelerate or interrupt a propagation process is one of the main research interests in complex network analysis. A correct spreader identification is a core need in applications, such as marketing, rumor spreading, identifying target points in power grids or main streets, preventing connectivity failures in large networks, such as Cellphone, Telephone, and the Internet, and, even more importantly, helping to prevent the spread of diseases or pandemics [3].

The problem of choosing a set of relevant nodes to achieve the maximum spreading flow is defined as the Influence Maximization Problem [4]; its main open issue is identifying the smallest number of influential nodes, from which the diffusion leads to broad network coverage. However, identifying vital nodes is not a trivial task. First, each propagation problem may specify a different criterion to define a node as relevant. Second, graphs modeling real and complex phenomena have nontrivial statistical and topological properties that do not occur in simple networks (lattices or random networks). Thirdly, most of the existing methods in the literature are designed to optimize the efficient dissemination of a single node, and not a set of nodes, although the latter is more relevant in real applications, e.g., in a spreading disease scenario, the aim is to immunize a group of users and not just one. However, it has been shown that selecting two or more good independent spreaders does not guarantee having the best set of spreaders as their influences may overlap [5].

The main approaches in the literature to identify influential nodes in a network come from three fields: operations research, evolutionary computation, and network science. Approaches from operation research aim to find a set of spreaders based on natural greedy strategies evaluated on independent cascade and linear threshold models [6]. From the evolutionary computation perspective, many algorithms have been recently proposed, either heuristics with provable guarantees (general greedy, high degree, and single discount) or metaheuristics, such as Evolutionary Algorithms (essentially genetic algorithms) [7,8]. In network science, distinct centrality indices have been proposed to address the identification of relevant nodes in a network. The most used metrics can be classified into three types: local, global, and random-walks [5,9]. Most known methods to identify relevant nodes use only structural information, which allows their application to phenomena that are different from those considered in their design [5]. In addition, the design of methods for some special types of networks, such as spatial, temporal, and multilayer networks, is a novel task in this research domain [6,10].

In social networks, as in other complex phenomena modeled as complex networks, the organization of the vertices, i.e., the topology of the network, plays a more prominent role in the identification of spreaders than a simple evaluation based on nodes connectivity. For example, a highly connected node located on the network boundary plays a less relevant role than a node with few connections but placed in the network core. By decomposing a network with the *k*-shell decomposition method, Kitsak et al. [11] showed that the node diffusion spread is affected by its network location. They found that the most influential nodes, namely the network core, could be identified in the largest *k*-core values.

In general, approaches designed to identify individual spreaders do not perform well in choosing a larger set of spreaders. Therefore, identifying a set of relevant spreaders for real phenomena modeled in large complex networks is still a significant, yet difficult, challenge.

In this paper, we propose a simple yet effective method that takes advantage of the underlying graph topology to guarantee that the selected nodes are not only relevant (efficient spreaders) but also are well scattered among the whole graph, which, according to our experimentation, allows us to obtain better diffusion values.

Our proposal is composed of two phases: first, a graph partitioning using a community detection algorithm; and second, the identification and distribution of relevant nodes for each community. Taking full advantage of induced partitions from the intrinsic clusters of the graph enhances the scatter of the relevant nodes allowing their distribution proportionally to the group sizes. With our proposal, the number of relevant nodes assigned per community is directly proportional to its size. Moreover, the number of communities can be used as a starting point to define a suitable number of spreaders since selecting the best spreader from each community helps to reach a wider network coverage.

The main contribution of this work is our strategy called Partition-Based Spreaders Identification (PBSI), which allows us to identify and scatter multiple influential spreaders more accurately than a relevant set of benchmark methods reported in the literature. The main advantages of our proposal can be summarized as follows: (1) It is computationally efficient, which makes it suitable to be applied to large real systems; (2) it helps to define a suitable number of nodes to be included in the set of spreaders; and (3) our proposed scattering strategy has been shown to improve most of the existing metrics.

## 2. Related Work

Many classic centrality methods can be used to characterize and classify a set of propagators in a complex network. Local metrics based on the node degree are simple and efficient, but they are the least effective, as they omit the overall structure of the network. On the other hand, global metrics, such as betweenness centrality and closeness centrality, perform well in identifying relevant spreaders but are highly computationally complex, making them unsuitable for large-scale networks [12,13]. Metrics based on Random walks, such as PageRank, ProfileRank, LeaderRank, and SpreadRank, have shown significant performance; however, information diffusion processes are not only based on random walks, as described in Reference [14].

It is worth mentioning that the influence capacity of the nodes is largely affected by their topological position. In this regard, the k-shell decomposition method performs a decomposition analysis selecting the spreaders set from nodes located within the network core. The k-shell method is found to be better than many local centrality metrics in many real networks. However, nodes within the same shell often have distinct influences. Moreover, nodes in the core-shell commonly show the rich club phenomenon, i.e., they are close together, and their influence is highly overlapped [15].

Several methods have been proposed to improve the *k*-shell decomposition. Zeng and others proposed a modification of the k-shell method called the mixed degree decomposition (MDD). Their method is based on a degree decomposition using the information of both residual degrees (number of links connected to the remaining nodes) and exhausted degrees (number of links connected to the removed nodes).

An approach to improve the performance of the *k*-shell decomposition algorithm is presented by Liu et al. [16]. They describe an efficient method to find the real influential spreaders based on link diversity of shells to distinguish the true core and core-like group. Liu et al. provide a method for improving the *k*-shell centrality by removing the redundant links that lead to densely connected core nodes, but they have low diffusion importance. The redundant links are identified by measuring the diffusion importance for each edge based on the number of out-leaving links of both ends. Ma et al. [17] proposed a gravity centrality index to identify the influential spreaders in complex networks. The authors use the idea of Newton’s gravity formula to measure the influence of nodes. Given two nodes v,w, and the value of a calculated metric $m_{v},m_{w}$, the metric values $m_{v}$ and $m_{w}$ are used as the masses in the gravity formula, and the shortest path between *v* and *w* is substituted as the distance value in the gravity formula. Authors apply this adapted gravity formula to achieve their graph ranking.

Zhang et al. [18] presented an iterative method to select a set of decentralized spreaders with high spreading capacity. In their method, called VoteRank, the influential spreaders are elected one by one according to voting scores obtained from their neighbors. In each iteration, all neighbors cooperate in the voting round with a percentage of their vote. The voting percentage of a node is reduced if one of its neighbors is selected as relevant.

More recently, Ahajjam et al. [19] proposed the HybridRank method to detect the influential spreaders in the network. Their method is based on the combination of two centrality metrics, Improved Coreness and Eigenvector, to obtain a hybrid value. Similarly, Yu et al. [20] presented an influential spreading method based on node indirect spreading. Their proposal considers indirect infections in the neighborhood of the nodes with a traditional direct infection strategy. However, in their proposal, to rate the indirect contribution of nodes, an alpha value must be defined for each network through a computationally expensive procedure, making the strategy unfeasible for large networks.

Lastly, Wang et al. [15] proposed an improved k-shell strategy called IKS. Their method employs a combination of node information entropy and k-shell decomposition to identify influential spreaders. Information entropy is used as a ranking metric, and the k-shells are used as a grouping strategy.

Although it has been found in the literature that the spreading process is dependent on the network topology, usually, spreading and choosing influential nodes are addressed independently. The methods mentioned above only rely on the computed metrics to rank and select the set of influential nodes. However, they do not consider that selected nodes can have a high relationship between them, causing a waste of resources/time influencing each other and not the rest of the network. Therefore, the strategy to select the set of relevant nodes must consider both their ranking value and their topological location.

## 3. Proposed Method

In general, an unweighted simple network is represented as G(V,E), where *V* is the set of vertices (or nodes), *E* is the set of edges (or ties) connecting some vertex pairs in *V*, *n* is the number of vertices n=|V|, and *m* is the number of edges m=|E|. In this section, we firstly introduce our proposal to enhance the ranking performance, and then several frequently used centrality indices and their potential applications are described.

Our PBSI method can be split into two processes: (1) the identification of influential spreaders using the gravity *k*-shell centrality and (2) the identification and scatter of a set of nodes that are susceptible to maximize the propagation of influence when working together. The details of our algorithm are described as follows.

### 3.1. Measuring the Relevance of the Vertices

The first step combines the improved version of the *k*-core centrality and the partitioning induced by a non-overlapping community detection algorithm to generate ranking values. Let $G=(V_{G},E_{G},C)$ be an undirected graph in which every vertex has been labeled with a community identifier by a community detection algorithm. Let us consider that *C* is the set $\left\{c_{1},c_{2},\ldots ,c_{nc}\right\}$ of non-overlapping communities identified in the graph.

The measurement of the relevant vertices is carried out for each community, i.e., a list of ranked vertices is obtained per each community of the graph. To rank the vertices, we use the Gravity *k*-Shell metric that is defined as follows:

(1) $$GKSW\left(v\right)=\sum_{w\in \psi_{3}}\frac{ks(v)\times ks(w)}{d_{vw}^{2}},$$

where ks(v) is the *k*-shell value for node *v*, $\psi_{3}$ are all the neighbors of *v* (without considering any structural partitioning) at most three steps away, and $d_{vw}$ is the shortest path length between *v* and *w*. The *k*-shell decomposition method was performed as defined by Batagelj and Zaversnik [21] and Kitsak et al. [11].

Equation (1) allows discriminating between the nodes of a graph, ranking them according to the value obtained for the gravity *k*-shell weighted metric. Once the number of spreaders per community has been defined, relevant spreaders can be selected and labeled accordingly to the ranking generated by the process described above.

### 3.2. Spreaders Distribution

As pointed out by Kitsak et al. [11], the range of propagation can be greatly improved if the set of selected spreaders are less connected between them (or also disconnected), especially when compared with a simple set ranked according to a centrality metric that does not consider the dispersion of the nodes to grant the ranking values.

In order to maximize the spread of influence, we propose to select the desired set of relevant nodes from each partition proportionally to the size of the community. Based on those assumptions, the separation of relevant nodes could accelerate the dissemination of information, and the selection of spreaders by the communities can lead us to affect as many nodes as possible. As in step one, the selection and distribution of spreaders are performed for each community. However, in the allocation, the communities are processed in size-order, from the smallest to the largest one.

Given *P* number of spreaders, the allocation is performed iteratively, assigning at least one spreader for small communities and a larger number for the bigger ones. If a community is small, it can be efficiently influenced by a single relevant node; however, it becomes evident to use more than one spreader to influence larger communities that show more complex structures.

Let us consider that we have to allocate *P* spreaders among a set *C* of communities. Let $c_{u}$ be the community with the less number of vertices, and let $n_{u}$ be the total number of vertices in all the unallocated communities. The number of spreaders allocated to $c_{u}$ is computed by the following equation.

(2) $$p_{c_{u}}\leftarrow \max\{1,\left\lfloor P_{r}\times \frac{|c_{u}|}{n_{u}}\right\},$$

where $P_{r}$ is the number of unallocated spreaders, $|c_{u}|$ is the size of the community *u*, and $n_{u}$ is the number of vertices of the graph which has not been considered by the spreader allocation process.

Let $G=(V_{G},E_{G},C)$ be a graph in which every vertex has been labeled with a community identifier by a community detection algorithm. Let us consider that *C* is the ordered set $\left\{c_{1},c_{2},\ldots ,c_{nc}\right\}$ of communities in increasing order by community size. Assuming that we want to distribute *P* spreaders over the |C| communities, we wish to do that considering the following:

1. If a community is small, then we assign one spreader.
2. The number of spreaders assigned to a community is proportional to its size.

At the beginning of the process, $c_{u}$ is equal to the smallest community that has not been allocated some spreaders, i.e., $c_{u}$ corresponds to $c_{1}$. Similarly, $n_{u}$ is equal to $|V_{G}|$ because no vertex has been processed yet. Likewise, $P_{r}$ is initialized with *P* because no spreader has been allocated yet. Then, for each community $c_{u}\in sorted\left(C\right)$, $p_{c_{u}}$ is calculated, and the $p_{c_{u}}$ nodes with higher rank value from than community are selected. After each allocation, the values of $n_{u}$ and $P_{r}$ are updated using: $n_{u}\leftarrow n_{u}-\left|c_{u}\right|$ and $P_{r}\leftarrow P_{r}-p_{c_{u}}$.

Following the described process, all the *P* spreaders are allocated through all the communities of a given graph, guaranteeing at least one spreader and that the number of allocated spreaders are proportional to the community size. Of course, to fulfill the above description, *P* should be greater than |C|.

When *P* is smaller than |C|, the communities are sorted in descending order (by community size), and the nodes with higher ranking value for the first |P| communities are selected as spreaders. When all communities have been processed, the graph with the allocated spreaders is returned.

## 4. Evaluation Design

To evaluate our proposal, we build a prototype that performs all tasks presented in Section 3 by using the python programming language and the python-igraph3 library. In addition, we built prototypes for the set of benchmarks methods presented in the following sections (Our code is available at: https://github.com/jedidiah-yanez/PB-Spreaders-Identification) (accessed on 9 September 2021).

Each method was evaluated using the SIR diffusion model by assessing three performance metrics. The experimentation was carried out using a set of nine real complex networks of increasing size to evaluate the quality of the selected diffusers, as well as the scalability of the methods.

### 4.1. Benchmark Methods

The following is a description of the benchmark methods that will be used to compare with our proposal. For each method, its name and the acronym used across the following sub-sections are given.

#### 4.1.1. Degree Centrality (DEG)

It is the degree of a vertex. Degree weighs the vertex relevance based on the number of links that it has. For directed graphs, two different metrics can be defined corresponding to the vertex incoming and outgoing degree. Degree centrality is an indicator for local spreading, and their influence is limited to their direct environment [22,23].

#### 4.1.2. Closeness Centrality (CLO)

Closeness centrality measures the closeness of one user to all other users. The closeness centrality of vertex *u*, Cc(u), is inversely proportional to the sum of the distances of *u* to every other vertex in the graph.

(3) $$Cc\left(u\right)=\frac{1}{\sum_{v\in V}d_{uv}}.$$

If a vertex has a high closeness centrality, then that vertex can reach more easily other vertices on the graph, i.e., the vertex is closer or near to others. Closeness centrality is usually positively associated with other measures, such as degree, because it gives higher values to more central vertices, i.e., those with shortest-path length [22,24].

#### 4.1.3. Betweenness Centrality (BET)

It is a measure that quantifies the frequency or number of times a vertex participates as a bridge along the shortest path between two other vertices and is defined as:

(4) $$Bc\left(u\right)=\sum_{v,w\in V,v\neq w}\frac{\sigma (u,v,w)}{\sigma (v,w)},$$

where σ(u,v,w) is the number of shortest paths between *v* and *w* that pass through *u*, ∀v,w∈V,v≠w; and σ(v,w) is the total number of shortest paths between *v* and *w*, ∀v,w∈V,v≠w. In a real-world network, the greater the number of shorter paths in which a vertex participates, the greater its importance. Additionally, a similar metric can be applied to the graph edges, measuring the proportion of shortest paths that pass through an edge [22,23]. Betweenness centrality not only takes into account the direct relationships but also the indirect ones, thus being indicators for global relevant nodes.

#### 4.1.4. VoteRank Method (VR)

It is a heuristic proposed by Liu et al. [18]. The VoteRank method brings to each node the ability to vote. In each round, the node that receives the most votes from its neighbors is selected as influential and does not vote in subsequent rounds. The voting ability $va_{v}$ of nodes that vote for the selected influential spreader is decreased. The voting score $vs_{v}$ of node *v* is determined by its neighbors as $vs_{v}=\sum_{u\in \delta_{v}}va_{u}$. In each round, the node with the largest voting score is selected as a relevant, and the voting ability of all its neighbors $u\in \delta_{v}$ is weakened by a factor $f=1/\langle k\rangle$, where $\delta_{v}$ are the neighbors of *v*, and $\langle k\rangle$ is the average degree. Spreaders are selected sequentially until the desired number is reached.

#### 4.1.5. HybridRank Centrality (HC)

This heuristic method is proposed by Ahajjam et al. [19]. The HybridRank strategy is divided into two stages: First, the hybrid centrality given by $HC_{v}=ICC_{v}\times EC_{v}$ is computed for each node. This method uses the Improved Coreness Centrality (ICC) and the Eigenvector Centrality (EC). Second, to avoid the selection of adjacent neighbors when selecting the relevant nodes, after the first spreader is selected, their adjacent neighbors are removed from the ranked list, and the next spreader is the one with the highest rank in the remaining list.

#### 4.1.6. Indirect Spreading (SC)

This measure is proposed by Yu et al. [20]. The SC metric considers the direct and indirect infection spreading into account. Authors define the spreading strength $c_{ij}$ of an edge $e_{ij}$ as

(5) $$c_{ij}=1+k_{j}^{out}{\left[1+\frac{|D_{ij,2}|}{2^{2}}\right]}^{\alpha },$$

where $k_{j}^{out}$ is the number of edges $\left\{e_{jl}|l\notin \delta_{i}\;and\;l\neq i\right\}$, i.e., node *l* lies outside of the neighborhood $\delta_{i}$ of node *i*. $D_{ij,2}$ is the number of paths from node *i* and node *j* whose length are 2, and the parameter α serves to tune the contribution of $1+|D_{ij,2}|$ to the direct and indirect infection spreading from node *i* to node *j*. The spreading strength of node *i* is defined as

(6) $$SC_{i}=\sum_{j\in \delta_{i}}c_{ij}.$$

#### 4.1.7. Improved K-Shell (IKS)

This method is proposed by Wang et al. [15]. Having $k_{i}$ as the degree of node *i*, the node information entropy is defined as

(7) $$e_{i}=-\sum_{j\in \delta_{i}}\frac{k_{j}}{\sum_{h=1}^{N}k_{h}}\cdot ln\left[\frac{k_{j}}{\sum_{h=1}^{N}k_{h}}\right],$$

where $\delta_{i}$ is the set of neighbors of node *i*, and $k_{j}/\sum_{h=1}^{N}k_{h}$ characterizes the importance of node *i*. The IKS method is conducted using the following procedure: (1) Decompose the network into shells by the k-shell decomposition algorithm; (2) for all nodes, compute the node information entropy, according to (7); (3) for each shell, sort nodes descending according to their information entropy; (4) for nodes in the highest k-shell value, select the node which has the largest information entropy, and then select the node in next to the highest shell which has the largest node information entropy; (5) repeat Step 4 and select the residual nodes until all nodes have been selected. When the value of the node information entropy is equal in the specific shell for two or more nodes, randomly choose one of them.

### 4.2. Spreading Model

A way to evaluate the spreading capabilities of a set of nodes is by using a spreading model. To evaluate our method, we apply the standard susceptible-infected-recovered (SIR) model, which has been extensively studied in many epidemics spreading processes [3,25]. In the SIR model, a node may belong to one of three states: Susceptible (S), Infected (I), and Recovered (R). The susceptible state (S) represents nodules that are likely to be infected but have not yet been infected. Infected (I) denotes nodes that can infect their susceptible neighbors. Recovery (R) involves individuals who were infected but have recovered and will never be re-infected.

At the beginning of the simulating process, all nodes are in susceptible status, except for an initial set of ρ infected nodes selected as source spreaders. At each time step, each infected node randomly contacts a neighbor and transmits the disease to it with probability β, if the latter one is susceptible. At the same time, each infected node will get recovered with a probability of μ. For generality, in this paper, we set μ=1.

The process terminates if there is not any infected node in the network. The final spreading scope $F_{SS}$ of the initial set ρ of spreaders is computed by counting the number of recovered nodes over 100 simulations. We set the value of infection probability β to be slightly larger than the network epidemic threshold $\beta_{th}\approx \frac{\langle k\rangle }{\langle k^{2}\rangle }$, where $\langle k\rangle$ is the average degree [3].

### 4.3. Performance Metrics

We use three metrics to evaluate the performance of tested methods. The first two are based on the spreading scale under SIR spreading model, and the third is based on the structural properties of selected relevant nodes.

In order to compare the *spread level* for different methods at distinct moments of the SIR simulation, we use the Spreading Scope SS at time *t* defined as:

(8) SS(t)=|ρ|+I(t),

where ρ is the set of nodes that are initially in the infected state, and I(t) is the number of newly generated infected nodes at time *t*.

To compute the *final scale* of affected nodes, we use the Final Spreading Scope $F_{SS}$, which is defined as:

(9) $$F_{SS}=\left|\rho \right|+I,$$

where ρ is as above, and *I* is the number of newly generated infected nodes when the spread process achieves the steady-state.

To evaluate the *structural properties* between each pair of selected relevant nodes, we compute the average shortest path length $L_{S}$, defined as:

(10) $$L_{S}=\frac{1}{\left|V|(|V|-1\right)}\sum_{\begin{array}{c} u,v\in V\\ u\neq v \end{array}}d_{u,v},$$

where $d_{u,v}$ is the length of a shortest path from node *u* to *v*.

### 4.4. Data Description

To verify the performance of our proposal, we have applied it to nine real networks with different sizes that are commonly used for efficiency comparison. The involved networks are the following: *USAir*, American aviation network [26]; *NetSci*, Co-authorship network of scientist [27]; *Email-EU-core*, core of the email data from a European research institution [28]; *PGP*, Confidential communication network using the Pretty Good Privacy encryption algorithm [29]; *CondMat*, represents Arxiv Condense Matter Physics collaboration network [30]; *Email-EU*, email data from a European research institution [28]; *Amazon*, network collected by crawling Amazon website [31]; *DBLP*, co-authorship network where two authors are connected if they publish at least one paper together [32]; and YouTube, on the YouTube social network, where users form friendships with each other, and users can create groups that other users can join [32]. For the definition of the community structure of each real network, the multi-level modularity optimization algorithm, also known as the Louvain algorithm, was applied [33].

Table 1 summarizes the main characteristics of the nine networks. As shown in the table, the networks are horizontally divided into three groups according to their size, ranging from hundreds of nodes and thousands of edges in the first group to millions of nodes and edges in the third group. For simplicity, these networks are treated as undirected and unweighed networks in this work.

## 5. Results and Discussion

We compared the performance of the proposed approach PBSI with the benchmark methods described above using the previously mentioned performance metrics. The evaluation was carried out by applying the SIR model and calculating the three metrics to the real networks. First, a set of relevant nodes is selected by using each benchmark method. Then, using each set of selected nodes as source spreaders, the spreading simulation is performed according to the SIR model described above.

**Figure 1:**
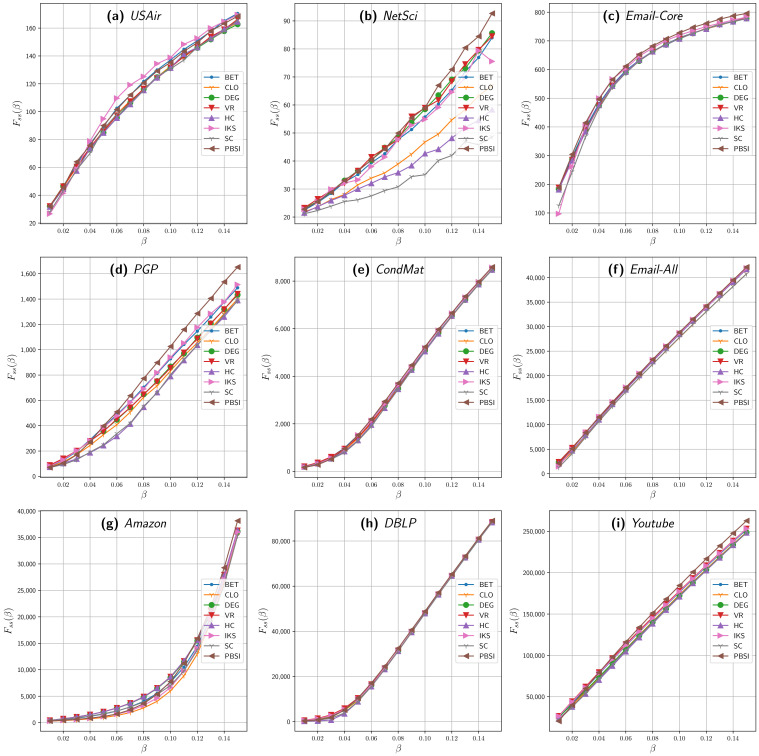
The final spreading scope for PBSI and benchmark methods on nine real networks when varying the spreading rates (β). The number of initial source spreaders ρ are defined as follows: ρ=20 for (**a**,**b**); ρ=50 for (**c**,**d**); ρ=100 for (**e**,**f**); ρ=200 for (**g**,**h**); and ρ=10k for (**i**). SIR model with μ=1. The influence spread ($F_{SS}$) on the y-axis against propagation probability β on the x-axis. Acronyms are defined in Section 4.1.

Figure 1 shows the final spreading scope $F_{SS}$ achieved by the different methods on the nine networks for different spreading rates β, from 0.01 to 0.15. For the first two groups of real networks (small and midsize), the SIR process was repeated 500 times using each method, achieving results reliability and reducing computation time. For the last group, the SIR process was repeated 100 times, an average value that is used in the literature.

The results shown in Figure 1 reveal that the set of relevant nodes identified and assigned by our proposal achieved a higher influence value than their counterparts, reaching a higher number of nodes in a broad region of β, especially for β≥0.05. In the widely used complex network PGP (Figure 1d), the set of relevant nodes selected by the PBSI method can reach more vertices than any other almost for all the spreading probabilities. A comparable result can be seen for the NetSci (Figure 1b), Email-Core (Figure 1c), Amazon (Figure 1g), and YouTube (Figure 1i) graphs, in which our approach performs with similar results than betweenness centrality but with less computational effort, as it will be shown later. For Figure 1d,f,i, our proposal stands out from β=0.05. When β=0.15, our proposal achieves a final spreading scope of 42,145.62 for the Email-All network (Figure 1f), while the nearest benchmark method (IKS) reaches on average 41,923.84. Similar results are achieved for Amazon (Figure 1g) and YouTube (Figure 1i), two of the biggest analyzed networks. For Amazon, PBSI reached a spreading of 38,151 and 262,814.44 for YouTube, while its nearest contender averages a spreading of 36,384.78 (HC) and 253,233.46 (VoteRank), respectively.

When the propagation probability is low (e.g., β<0.04), the spreading initiated from selected nodes are more likely to be confined in local regions. In this case, the greater the degree of nodes, the more neighborhood would be directly activated since a high degree provides a greater chance to spread, i.e., methods based on degree would perform better than others.

**Figure 2:**
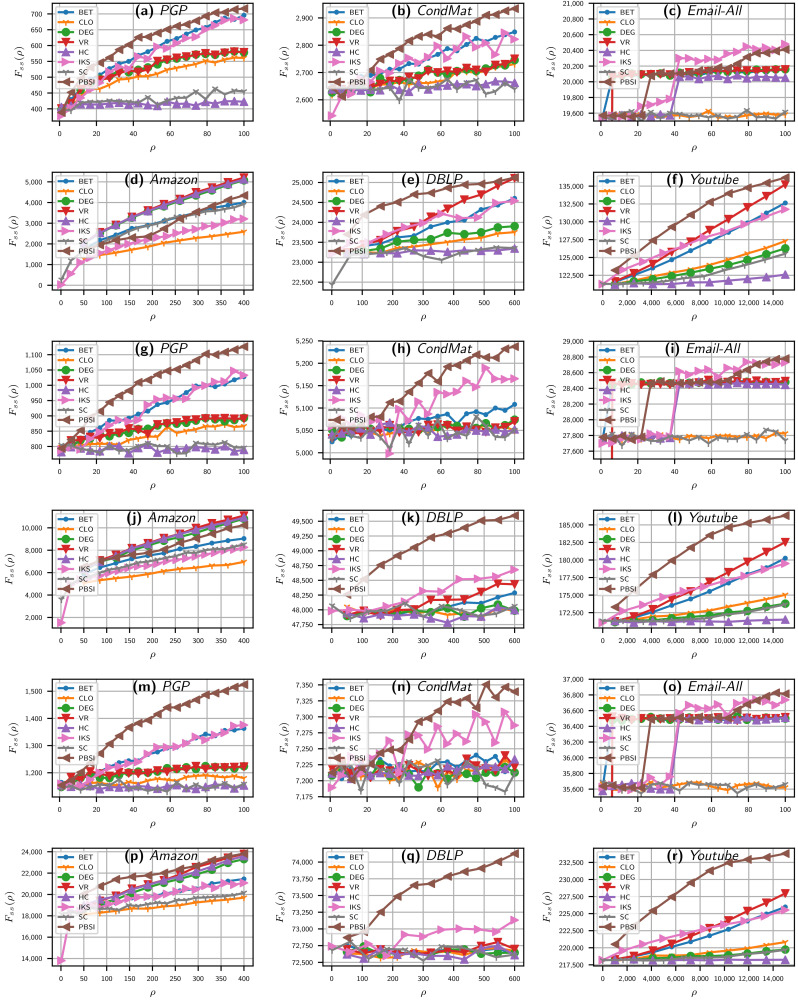
The final spreading scope for PBSI and benchmark methods on six real networks and three spreading rates (β) when varying the number of spreaders (ρ). SIR model with μ=1, ρ ranges up to 100 in PGP, CondMat, and Email-All, up to 400 and 600 in Amazon and DBLP, and up to 15k in the YouTube network. Plots (**a**–**f**) were computed with β=0.07, plots (**g**–**l**) with β=0.10, and plots (**m**–**r**) with β=0.13. ρ spreaders in *x*-axis against $F_{SS}$ in *y*-axis. Acronyms are defined in Section 4.1.

In Figure 2, the effects of varying the number of spreaders ρ for the benchmarks methods and PBSI using three different information transmission rates (β) are reported. In the *x*-axis, the ρ spreaders are plotted, and, in the *y*-axis, the $F_{SS}$ value for each given ρ is plotted. Figure 2 is composed of three groups of each one of the six plots. Each group shows the SIR results for the real networks of mid and large sizes using a distinct value of β, namely 0.07, 0.10, and 0.13. The first six plots (Figure 2a–f) show results for β=0.07; the next group of plots (Figure 2g–l) shows results for β=0.1, and last group (Figure 2m–r) shows results for β=0.13.

From Figure 2, it can be seen that PBSI has an advantage over the reference methods for a wide rho region in the three beta values analyzed, mainly in the PGP, CondMat, DBLP, and YouTube networks. As can be seen in all the plots, the influence spread of our approach continues to grow with an increase of ρ in all networks. In cases, such as the PGP and CondMat networks, the influence spread of degree heuristic almost ceased to grow, and their result curves are nearly parallel to the *X* axis (mainly for β equal to 0.1 and 0.13). This shows that the source nodes selected by traditional heuristic algorithms tend to have larger overlaps, which leads to the redundancy of propagation [34].

**Figure 3:**
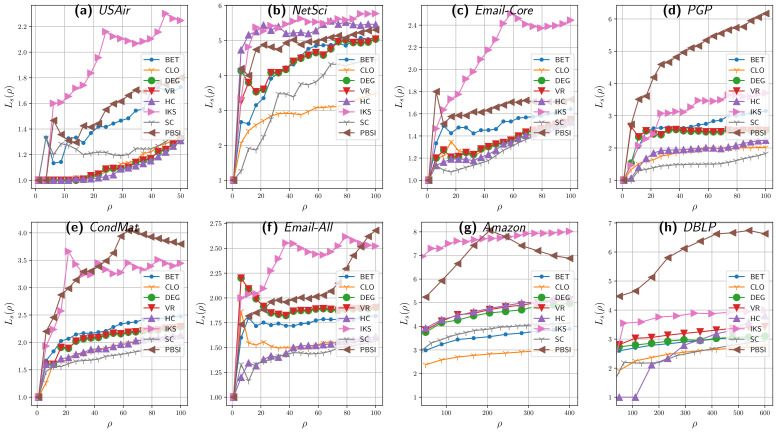
The average shortest path length $L_{S}$ among the source spreaders selected by PBS and the benchmark methods on eight real networks when varying the number of spreaders (ρ). ρ ranges from 1 to 50 in USAir (**a**), 1 to 100 in the next five networks (**b**–**f**), 50 to 400 in Amazon (**g**), and 1 to 600 in DBLP (**h**). The average shortest path length on the *y*-axis against the number of multiple spreaders ρ on the *x*-axis. Acronyms are defined in Section 4.1.

To verify the effectiveness when scattering the relevant nodes selected by the PBSI metric compared to the scattering achieved by benchmark methods, we evaluate the average shortest path length metric $L_{S}$. As can be seen in Figure 3, the selected influential spreaders obtained using the PBSI method have larger $L_{S}$ values than most of the achieved by the benchmark methods, especially in the larger networks and for higher ρ values. Compared with the benchmark methods, the source spreaders selected by PBSI are more decentralized in the whole network. Actually, as described in Reference [35], the higher the values of $L_{S}$, the more effective the distribution. Due to the computational complexity of calculating all the shortest paths lengths in big graphs and for a large number of spreaders, Figure 3 shows only the average shortest path lengths for the first eight real networks presented in Table 1.

Figure 3 shows $L_{S}$ of source spreaders selected by different methods under different scales of source spreaders, 1 to 50 in USAir (Figure 3a), 1 to 100 for NetSci (Figure 3b) to Email-All (Figure 3f), 50 to 400 in Amazon (Figure 3g), and 50 to 600 in DBLP (Figure 3h). It can be observed that, in the three smaller networks, the distances between each pair of spreaders achieved by the proposed method are smaller than the ones obtained by IKS and HC metrics, but they are greater than all the other applied methods. In addition, it can be seen in Figure 3 that, in most of the cases, the $L_{S}$ value achieved by PBSI has three sections: first, an increasing behavior, then a slight descent, and, finally, it recovers its increasing trend. This behavior is associated with the distribution strategy. When ρ≤|C|, the spreaders are assigned to each community, reaching the maximum distribution value ($L_{S}$) when ρ=|C|. For the cases when ρ≥|C|, the spreaders are assigned proportionally to the community sizes. Having more than one spreader per community reduces the average distance between spreaders of the same community, reflecting a slight decrease.

This trend can be observed in Amazon (Figure 3g), which has 214 communities. In Amazon’s case, we can observe the growing trend for values of ρ≤214, the peak when ρ=214, and a decreasing trend for the subsequent values. Less noticeable, the DBLP’s case in (Figure 3h), with 573 communities, shows the increasing trend until ρ≤573, a slight peak when ρ=573, and a less abrupt drop for later values. CondMat (Figure 3e), having 62 communities, shows the same behavior. In the cases of PGP (Figure 3d) and Email-All (Figure 3f), only the increasing trend can be observed since the number of communities, 117 and 115, respectively, are outside the plotted range.

Despite the above, it can be observed that the trends obtained by PBSI show a better profit of $L_{S}$ regarding the benchmark methods in the whole analyzed ranges. In summary, the results of the average shortest path length metric, in combination with the results of the SIR simulation experiments, show that our method not only improves the diffusion from the selected source nodes but also ensures that they are well scattered.

Finally, we test the profit generated by the distribution strategy (Step 2 of the proposal) when it is combined with the benchmark methods. As mentioned above, taking advantage of the community partitioning not only helps to guarantee a good vertex distribution but also contributes to use the number of communities as a guide to define the number of relevant nodes that can have a good spreading of the information. For this experimentation, the number of spreaders was set as the number of communities obtained by Louvain’s algorithm [33], i.e., ρ=|C| (last column of Table 1).

The SIR simulation was performed using μ=1 and β=0.13. Each reference method was calculated in its simple form and using the distribution scheme of our proposal, i.e., using the communities as a base and calculating the values of the method for each community. Finally, for each community, the vertex with the highest value according to the selected metric was used.

In the columns of Table 2 the results are shown for eight of the nine real networks used. In turn, rows show the results obtained by each method. The highest value for each network is shown in bold. The combination of our distribution proposal with each benchmark method is marked with the symbol “⋆”.

In addition to the results obtained for the benchmark methods, in the table, we include the results achieved when selecting the spreaders based on the node’s True Spreading Ability (TSA). The TSA of each node is calculated by evaluating the SIR model using that node as a spreader. The ranking based on TSA is considered in the literature as the ideal ranking; therefore, in several works, it has been used as a base to perform correlation evaluations of the rankings generated by other strategies. However, as shown in the results of Table 2, the spreaders selected using the TSA strategy are far from reaching the best diffusion values.

As shown in the table, almost all the diffusion values achieved by the benchmark methods improve when combined with our distribution strategy. Furthermore, the DEG method, which is considered the simplest and least relevant, overcame the diffusion achieved by nearly all methods in its simple version when combined with our distribution strategy (DEG⋆) (only in the case of the two smaller networks this was not fulfilled).

Table 2 also shows that, in five of the eight evaluated networks, the maximum coverage value is reached using our strategy (cells in bold). For the remaining networks where PBSI does not achieved the highest value, the maximum coverage value was obtained by methods combined with our distribution strategy HC⋆, DEG⋆, and IKS⋆ for USAir, PGP, and CondMat, respectively. This shows that both the selection of relevant nodes and distribution strategy of our proposal overcome the results achieved by the benchmarks methods, which are commonly used in the literature.

Further, it can be seen that the spreading results achieved by TSA fail to improve any metric in any of the analyzed networks. Despite having evaluated all the nodes in the network and knowing their diffusion value, it can be observed that the Top-*C* ranked spreaders do not necessarily have the best performance.

**Figure 4:**
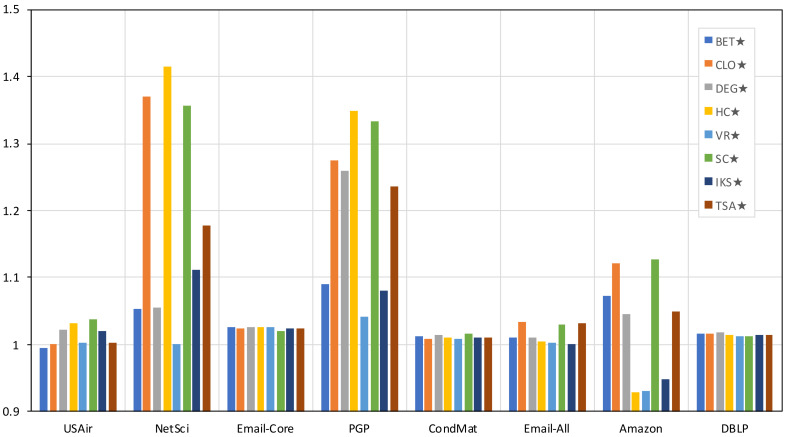
The profit margin achieved by the reference methods combined with the distribution strategy compared to their simple versions on eight real complex networks. Acronyms are defined in Section 4.1.

Figure 4 shows the percentage of profit (Δ) of the metrics combined with the distribution strategy compared to the simple metrics. These values were obtained by applying Δ(metric)=metric⋆/metric. If Delta(metric)>1 means a profit over the simple metric; on the other hand, if Delta(metric)<1, it means that the combined metric performed worse than the simple metric. Figure 4 shows that only in the USAir (BET⋆) and Amazon (HC⋆ and VR⋆) networks is a worse performance of the combined metrics observed. As can be seen in the figure, the highest gains were achieved in the NetSci and PGP networks, where a gain of more than 30% can be observed. In the figure, the greater the value above one, the greater the gain obtained by the combined metric. The average profit considering all the networks and methods is 5.95%.

Although the profit obtained from the combined schemes may seem low, it is necessary to point out that, except for USAir and NetSci, the profit obtained for the DEG⋆ metric was enough to exceed the results achieved by more complex and specialized metrics, such as BET, VR, HC, and IKS, as can be observed from Table 2.

### Computational Efficiency

The proposed method PBSI is composed of two steps: (1) the centrality calculation and (2) the identification of a set of influential spreaders in the networks. The computational complexity of the ranking method depends on the size of the neighborhood covered, which in our method is bounded by $O(|V|\times \langle k\rangle \times d)$, where $\langle k\rangle$ is the average degree of the network, and *d* is the order (ratio) of the neighborhood. We use d=3; therefore, the resulting time complexity for first step is $O(3\times |V|\times \langle k\rangle )$, which is dominated by $O(|V|\times \langle k\rangle )$, and this, in turn, for O(|V|) if considering that, in most complex networks, the average degree $\langle k\rangle$ is less than the number of nodes, i.e., $\langle k\rangle \ll \left|V\right|$.

The second stage, on the other hand, performs a sort operation based on the community sizes, having O(|C|log|C|) time complexity. Then, a sort of all nodes based on their ranking value is performed, and a scan for the nodes of each community is performed, i.e., O(|V|log|V|+|C|×|V|). As |C|≪|V|, the resulting time complexity is defined as O(|V|log|V|). Thus, the final time complexity is O(|V|+|V|log|V|), which is dominated by O(|V|log|V|).

**Figure 5:**
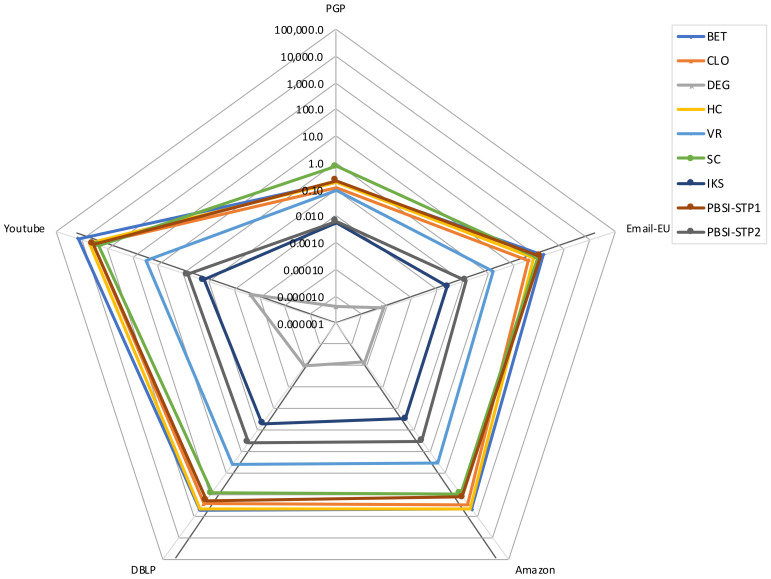
Time in seconds, consumed by the two steps of PBSI, and the benchmark methods for the mid-size and large-size networks. Axes are in log scale. On the five axes are the five networks, and each line corresponds to each evaluated method. Acronyms are defined in Section 4.1.

Figure 5 shows the execution times of the methods analyzed. Each axis depicts the time consumed for each method for a specific network. Axis are in log scale from the innermost value $1\times 10^{-6}$ to the outermost $1\times 10^{5}$. Correspondingly, lines describe the time consumed. The figure compares the time consumed by the two stages of our proposal PBSI regarding the time consumed by the benchmark methods when analyzing five of the largest networks presented in Table 1.

From the figure, it can be observed that the behavior of PBSI-STP2 (the distribution proposal) have a minor impact in time, as it only grows two orders of magnitude from the smaller graph (PGP) to the bigger one (YouTube), which matches to the size of the graphics, whose difference in the number of vertices and edges is two orders of magnitude. On the contrary, algorithms, such as BET, HC, and SC, have a temporary growth of more than four orders of magnitude for the same graphs in similar scenarios. The smallest amounts of time are achieved by DEG, IKS, and PBSI-STP2, as shown in the figure.

## 6. Conclusions

Identifying a set of relevant nodes to maximize the spread of influence in complex networks is an important task in areas, such as social network analysis and viral marketing. The most used strategy to solve this issue is to compute a simple centrality method and to select as relevant nodes the set of top-ranked nodes. Although many methods have been proposed in the literature to select relevant nodes from a complex network, most of them use the idea of ranking the vertices accordingly to a defined metric. However, when more than one node needs to be selected as a spreader, it has been shown that neglecting the network topology and using a greedy selection of the top-ranked nodes will not achieve the best spreading capabilities. Furthermore, we have found that even in the simple and computationally effortless method, the degree, can outperform some of the more complex metrics when the network topology is considered.

In this paper, we presented a methodology based on data science that aims to identify relevant nodes in a complex network according to the partitioning of a graph given by a ground truth or a community detection algorithm. In our method, the set of influential spreaders is chosen not only based on a centrality method but also proportional to the community sizes; the proposal allows the selected nodes not to be adjacent, thus maximizing the spread, even of existing centrality methods.

Our proposal is composed of two stages. First, the relevance of the vertices belonging to each induced partition is measured. Second, given an ρ number of relevant nodes to be selected, they are selected from the partitions and proportionally to their size. The selection of relevant nodes is performed based on two premises: (1) to select good spreaders and (2) to have a high scattering. The performance of our method was evaluated in nine real networks under the Susceptible-Infected-Recovered (SIR) model.

Experimental results for the nine processed networks in the SIR model show that the proposed method outperforms several benchmark methods using different metrics in both (1) spreading capabilities and (2) distribution of relevant nodes. Furthermore, our method to scatter the relevant nodes allowed us to improve the results obtained by the benchmark metrics used. Results confirm that it is possible to take advantage of the graph topology, i.e., the partitioning induced by the communities to achieve a good scattering of the relevant nodes.

In addition, we show that the complexity of our proposal makes it suitable to process large complex networks with lower computational effort when compared with other metrics, such as Betweenness centrality and HybridRank. 

## Figures and Tables

**Table 1:** Summary of basic topological properties of the 9 real networks. *V* and *E* are the total number of nodes and edges, respectively. $\langle k\rangle$ is the average degree. *L* is the average shortest path. $\langle Cc\rangle$ is the average clustering coefficient. $ks_{max}$ is the maximum *k*-shell value, and *C* is the number of communities identified in the graph.

Network	*V*	*E*	$\langle k\rangle$	*L*	$\langle \mathrm{Cc}\rangle$	${\mathrm{ks}}_{\max}$	*C*
USAir [26]	332	2,126	12.807	2.738	0.749	26	7
NetSci [27]	379	914	4.82	6.042	0.798	8	18
Email-EU core [28]	1,005	25,571	50.88	2.586	0.399	56	41
PGP [29]	10,680	24,316	4.55	7.463	0.440	31	117
CondMat [30]	23,133	93,497	8.51	5.352	0.633	25	62
Email-EU [28]	265,214	420,045	3.03	4.118	0.486	39	115
Amazon [31]	262,111	1,234,877	9.42	8.831	0.420	10	214
DBLP [32]	317,080	1,049,866	6.62	6.792	0.632	113	573
YouTube [32]	1,134,890	2,987,624	5.27	5.279	0.080	51	9635

**Table 2:** Comparison between the final spreading scope achieve by the simple benchmark methods and their version combined with our proposed scattering method, which distributes the relevant nodes according to the partitioning induced by a community scheme. The highest values for each graph are shown in bold. (+) means a higher value than the simple metric. (−) means lower value than the simple metric. Acronyms are defined in Section 4.1.

	USAir	NetSci	Email-Core	PGP	CondMat	Email-All	Amazon	DBLP
**BET**	155.04	66.75	756.28	1401.24	7221.25	36,498.84	19,928.94	72,673.86
**BET⋆**	154.19 −	70.28 +	775.36 +	1526.5 +	7311.28 +	36,852.86 +	21,368.11 +	73,896.33 +
**CLO**	153.75	53.10	755.08	1203.53	7221.54	35,627.50	18,996.47	72,647.98
**CLO⋆**	153.87 +	72.76 +	774 +	1533.98 +	7283.41 +	36,825.19 +	21,317.18 +	73,863.33 +
**DEG**	151.42	70.38	755.76	1242.64	7205.79	36,464.72	20,892.47	72,679.94
**DEG⋆**	154.78 +	74.21 +	774.91 +	**1565.75 +**	7307.24 +	36,863.84 +	21,825.99 +	74,039.21 +
**HC**	152.47	50.74	755.74	1157.59	7205.75	36,500.04	21,131.07	72,650.26
**HC⋆**	**157.49 +**	71.79 +	775.35 +	1562.12 +	7278.66 +	36,707.82 +	19,646.13 −	73,661.48 +
**VR**	153.28	76.04	755.63	1494.00	7224.53	36,581.93	21,176.56	72,753.74
**VR⋆**	153.66 +	76.06 +	775.69 +	1557.41 +	7284.29 +	36,672.45 +	19,709.25 −	73,621.61 +
**SC**	151.63	41.13	757.19	1147.16	7221.50	35,647.38	19,173.56	72,715.13
**SC⋆**	157.22 +	55.81 +	772.16 +	1529.56 +	7333.97 +	36,750.59 +	21,600.94 +	73,680.69 +
**IKS**	150.78	63.94	757.34	1392.66	7264.97	36,811.25	20,041.84	73,038.16
**IKS⋆**	153.72 +	71.13 +	776.06 +	1504.31 +	**7336.69 +**	36,845.56 +	18,993.88 −	74,100.79 +
**TSA**	153.97	51.91	758.44	1154.72	7197.31	35,597.56	18,806.53	72,612.16
**TSA⋆**	154.47 +	61.09 +	776.5 +	1427.03 +	7270.59 +	36,744.88 +	19,728.44 +	73,735.09 +
**PBSI**	156.10	**76.40**	**776.85**	1556.82	7316.78	**36,868.83**	**21,857.31**	**74,277.69**

## Data Availability

The complex networks analyzed in this study are publicly available. Datasets used in this paper can be found in references [26,27,28,29,30,31,32]. Paper’s code is available at: https://github.com/jedidiah-yanez/PB-Spreaders-Identification (accessed on 9 September 2021).
